# Primary Ηypoparathyroidism in a Patient With Sarcoidosis: A Case Report

**DOI:** 10.7759/cureus.69504

**Published:** 2024-09-16

**Authors:** Konstantinos Dodos, Vasileia Tsampika Kalamara, Vasiliki E Georgakopoulou, Paraskevi Kavoura

**Affiliations:** 1 Department of Respiratory Medicine, General Oncological Hospital of Kifissia “Agioi Anargyroi’’, Athens, GRC; 2 Laboratory of Physiology, Aristotle University of Thessaloniki, Thessaloniki, GRC; 3 Department of Pathophysiology/Pulmonologist, Laiko General Hospital, Athens, GRC

**Keywords:** calcium homeostasis, endocrine disorders, hypocalcemia, hypoparathyroidism, sarcoidosis

## Abstract

Sarcoidosis is a complex, multisystem granulomatous disorder with a variable clinical presentation, commonly involving the lungs but potentially affecting any organ system. This case report describes a rare occurrence of primary hypoparathyroidism coexisting with sarcoidosis in a 45-year-old male patient. The patient presented with a chronic cough and progressive breathlessness. Diagnostic imaging and histopathological evaluation confirmed stage 1 sarcoidosis, characterized by non-caseating granulomas and elevated angiotensin-converting enzyme (ACE) levels. Surprisingly, the patient also exhibited significant hypocalcemia, hyperphosphatemia, and low parathyroid hormone levels, which led to the diagnosis of primary hypoparathyroidism, an unusual finding in the context of sarcoidosis. The patient was treated with corticosteroids for sarcoidosis and calcium supplementation for hypocalcemia, resulting in symptom resolution and normalization of biochemical parameters. This case highlights the importance of considering multiple endocrine disorders, such as hypoparathyroidism, in patients with sarcoidosis, especially when calcium dysregulation is observed. The coexistence of these conditions presents unique diagnostic and therapeutic challenges, necessitating a multidisciplinary approach to patient care.

## Introduction

Sarcoidosis is a complex, multisystem granulomatous disorder of unknown etiology that can affect individuals of all ages and ethnicities, though it most commonly manifests in adults between 20 and 50 years of age. The pathogenesis of sarcoidosis is believed to involve an aberrant immune response in genetically predisposed individuals triggered by exposure to unidentified environmental antigens. This causes non-caseating granulomas to form. These are groups of activated immune cells, such as macrophages, lymphocytes, and epithelioid cells, that can get into and hurt different body organs [1].

The clinical presentation of sarcoidosis is highly variable, as the granulomas can potentially develop in any organ system. However, over 90% of cases show pulmonary involvement, often leading to symptoms like dyspnea, cough, chest pain, and wheezing. Radiographic findings may include bilateral hilar lymphadenopathy, reticulonodular opacities, and/or parenchymal infiltrates. Extrapulmonary manifestations are also common, with the skin, eyes, reticuloendothelial system, and musculoskeletal system being the next most frequently affected sites [2].

The diagnosis of sarcoidosis involves a comprehensive clinical assessment, laboratory testing, and histopathologic evaluation. Elevated serum angiotensin-converting enzyme (ACE) levels and hypercalcemia may provide supportive evidence, though these findings are nonspecific. In the absence of alternative causes, a definitive diagnosis necessitates the identification of non-caseating granulomas on a biopsy of an affected organ, typically the lungs, lymph nodes, skin, or another accessible site. Imaging modalities such as chest radiography, high-resolution computed tomography (HRCT), and 18F-fluorodeoxyglucose positron emission tomography (FDG-PET) can also aid in disease assessment and monitoring. The treatment of sarcoidosis is largely empiric and tailored to the severity and pattern of organ involvement. Patients who are asymptomatic or mildly symptomatic may benefit from a watchful waiting approach that includes periodic clinical and radiographic follow-up, given that spontaneous remission can occur in up to 60% of cases. However, for those with more severe, progressive, or organ-threatening diseases, immunosuppressive therapy is usually required [3].

The pathogenesis of sarcoidosis-associated hypercalcemia is complex and involves the dysregulated production of 1,25-dihydroxyvitamin D (calcitriol) by activated macrophages within the granulomas. Normally, the kidney tightly regulates calcitriol synthesis; however, in sarcoidosis, the granulomatous infiltrates can autonomously produce large quantities of this active vitamin D metabolite. Calcitriol's potent stimulatory effects on intestinal calcium absorption and osteoclast-mediated bone resorption mediate the resulting hypercalcemia [4,5].

The aim of this article is to present a rare case of primary hypoparathyroidism coexisting with sarcoidosis, explore the diagnostic and therapeutic challenges associated with this unusual combination, and highlight the importance of a multidisciplinary approach in managing such complex clinical scenarios.

## Case presentation

A 45-year-old male patient presented with a chronic nonproductive cough (two months) and progressive dyspnea during exertion. He had no further medical history and was, in general, with no notable health concerns. The clinical evaluation revealed no clear signs, as the chest auscultation was normal, and there were no signs of heart failure (e.g., edema in the lower extremities). The patient did not report any classic symptoms of hypocalcemia, such as muscle cramps, tingling, numbness in the extremities, or seizures. The physical examination did not reveal any signs consistent with hypocalcemia, such as Chvostek’s sign or Trousseau’s sign. Neuromuscular function appeared normal.

**Figure 1:**
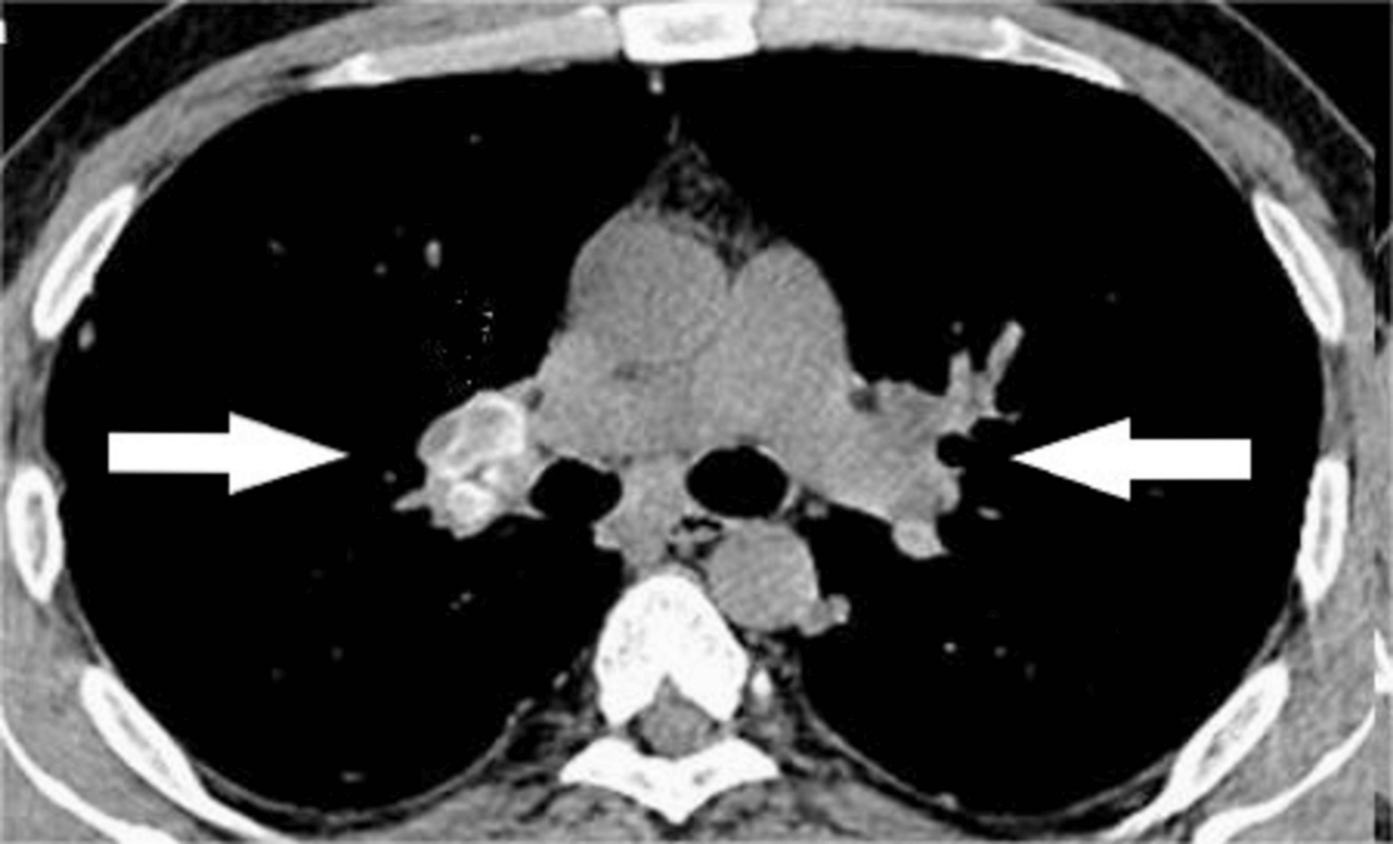
Chest computed tomography. Arrows show enlarged hilar lymph nodes.

The CT scan revealed enlarged hilar lymph nodes in both lungs, but there was no lung parenchymal involvement (Figure 1).

**Figure 2:**
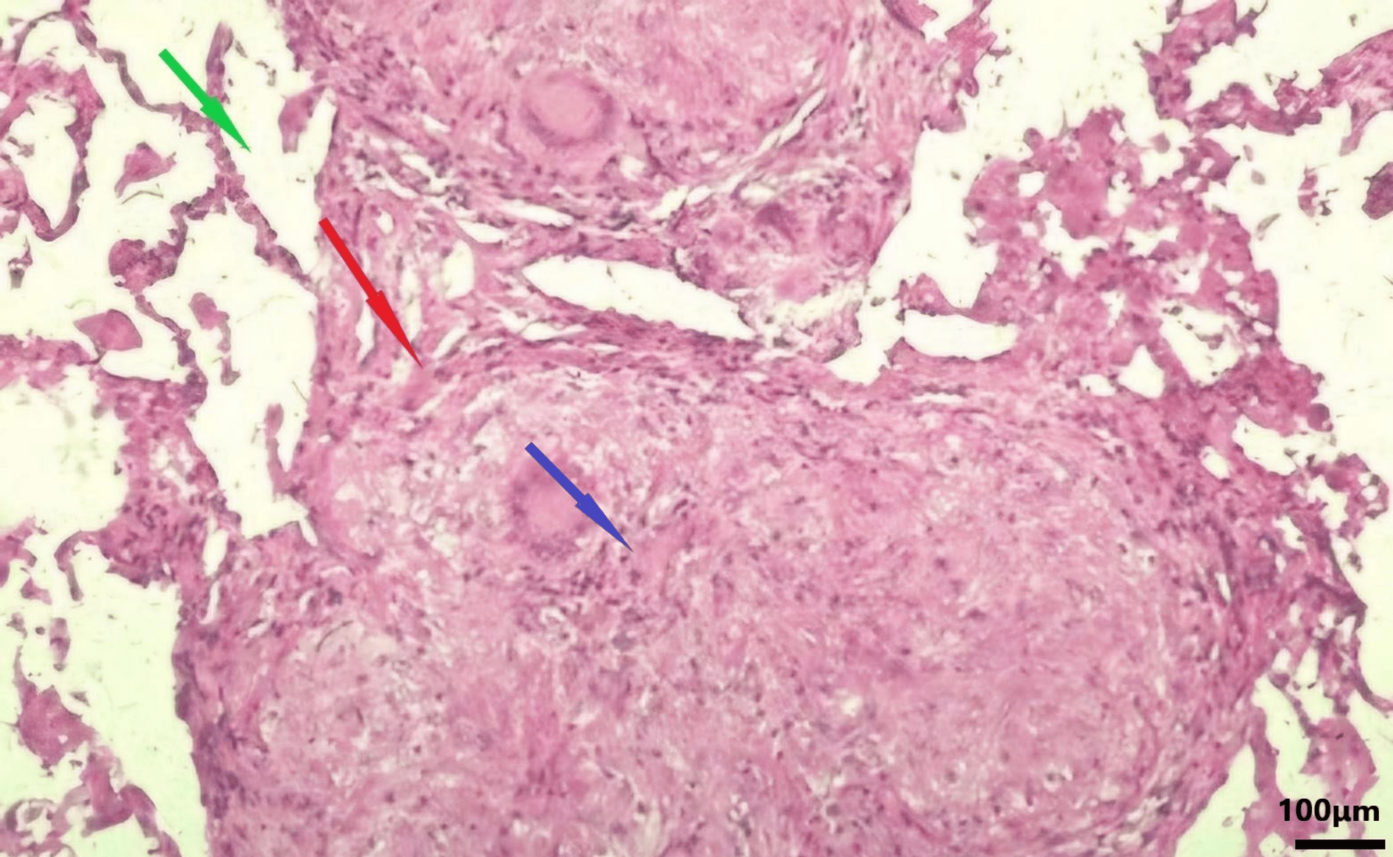
Histopathological image of the acquired biopsy (hilar lymph node). Histopathological section stained with hematoxylin and eosin (H&E); original magnification, ×20. Red Arrow points to granuloma: rounded, well-formed collection of epithelioid histiocytes; blue arrow points to a multinucleated giant cell; green arrow points to an area of fibrosis.

Following these findings, we performed an endobronchial ultrasound (EBUS) bronchoscopy on our patient and obtained biopsies. The histopathological evaluation of the tissue showed non-caseating granulomas, a typical finding in sarcoidosis (Figure 2).

The HRCT revealed no lung parenchymal involvement, indicating stage 1 sarcoidosis. Table 1 displays the results of the laboratory investigations.

**Table 1:** Laboratory investigations.

Parameter	Result	Normal range
ACE (angiotensin-converting enzyme)	79 U/L	8–52 U/L
Brain natriuretic peptide (BNP)	87 pg/ml	<100 pg/ml
Calcium	6.5 mg/dl	8.5–10.2 mg/dl
Ionized calcium	0.65 mmol/l	1.12–1.30 mmol/l
25-hydroxy vitamin D	42.1 ng/ml	30–100 ng/ml
1,25-dihydroxy vitamin D	40 pg/ml	18–72 pg/ml
Phosphate	7.5 mg/dl	2.5–4.5 mg/dl
Parathyroid hormone (PTH)	7 pg/ml	10–65 pg/ml
Magnesium	1.6 mg/dl	1.7–2.2 mg/dl

To clarify whether these abnormalities persisted, the calcium levels and relevant investigations were repeated. On re-evaluation, the serum calcium remained low, consistent with the initial findings. The patient did not have prior documented calcium levels before this presentation, so it is unclear whether this hypocalcemia developed recently or was chronic.

Since our patient did not report any neck-related surgical procedures, we can attribute all the above findings to primary hypoparathyroidism, a potential autoimmune etiology.

**Figure 3:**
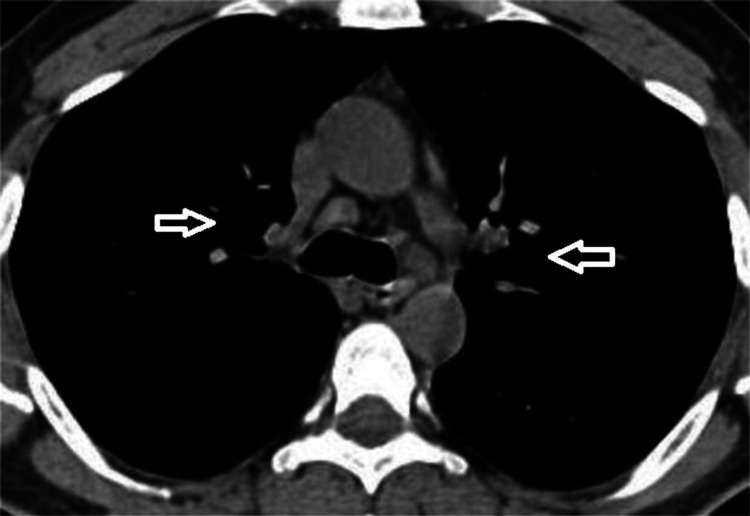
Chest computed tomography at three months. Arrows show the reduction in the diameter of both hilar lymph nodes.

As far as sarcoidosis treatment is concerned, prednisolone 0.5 mg/kg was prescribed, and our patient was put on follow-up. After three months of therapy, his symptoms started to subside, and his CT scan revealed a reduction in the diameter of both hilar LNs (Figure 3).

Corticosteroid therapy continued with a reduction in dosage for six months until complete resolution of symptoms and normalization of the CT findings. Due to the observed hypocalcemia, our patient was referred to an endocrinologist. The patient’s hypocalcemia was managed with calcium carbonate supplementation, a commonly used form of calcium for oral therapy. In addition to calcium supplementation, the patient also received active vitamin D (calcitriol).

## Discussion

To the best of our knowledge, this is a rare case of coexisting hypoparathyroidism with hypocalcemia and sarcoidosis. There are only four similar cases in the literature [6-9].

Zimmermann et al. [6] discussed a case of a 49-year-old woman with hypoparathyroidism, a condition typically characterized by low levels of calcium in the blood. Following a subtotal thyroidectomy, the patients in this case developed symptomatic hypocalcemia, which required treatment with vitamin D and calcium. However, after developing sarcoidosis, an inflammatory disease known to cause hypercalcemia through elevated levels of 1,25-dihydroxyvitamin D, her hypocalcemia unexpectedly resolved without the need for continued vitamin D or calcium supplementation. This case suggests that sarcoidosis can induce the synthesis of 1,25-dihydroxyvitamin D independent of PTH, potentially through the action of sarcoid granulomas.

Brinkane et al. [7] described a case involving a 52-year-old woman diagnosed with both hypoparathyroidism and sarcoidosis. Hypoparathyroidism emerged eight years after the initial diagnosis of sarcoidosis. No evidence supporting an autoimmune condition affecting the parathyroid glands was found, and both pathological and immunological blood tests were unremarkable. An alternative pathophysiological mechanism potentially linked to sarcoid granulomatous infiltration of the parathyroid glands was considered.

Saeed et al. [8] discussed a 27-year-old woman who presented with severe hypocalcemia shortly after giving birth. The patient exhibited symptoms such as fever, facial numbness, and tetanic spasms, along with other signs indicative of systemic illness. Further investigation revealed that she had both sarcoidosis, a disease known to cause granulomas and abnormal calcium metabolism, and DiGeorge syndrome, a genetic disorder associated with congenital heart defects and hypoparathyroidism. The patient responded well to treatment with calcium, vitamin D supplementation, and low-dose steroids.

Dill [9] discussed a 49-year-old woman who developed hypoparathyroidism after a subtotal thyroidectomy. For many years, she required vitamin D and calcium supplements to manage symptomatic hypocalcemia. However, she developed sarcoidosis, which unexpectedly resolved her hypocalcemia and eliminated the need for her ongoing supplements. This case is notable because it demonstrates that sarcoidosis can lead to elevated levels of 1,25-dihydroxyvitamin D independently of parathyroid hormone (PTH), correcting hypocalcemia in a patient with hypoparathyroidism.

In comparison to the cases reported by Zimmermann et al. [6], Brinkane et al. [7], and Dill [9], where patients with hypoparathyroidism developed sarcoidosis-related hypercalcemia that compensated for the hypocalcemia, our patient exhibited persistent hypocalcemia despite the coexistence of sarcoidosis. This highlights a different pathophysiological course, where granulomatous production of 1,25-dihydroxyvitamin D was insufficient to overcome the hypocalcemia. Unlike Zimmermann’s case, where sarcoidosis resolved the hypocalcemia, our patient required ongoing calcium supplementation, emphasizing the variable impact of sarcoidosis on calcium metabolism across different patients.

The clinical findings from this case underscore the critical importance of considering coexisting conditions such as hypoparathyroidism in patients diagnosed with sarcoidosis. This is particularly relevant given the well-documented association between sarcoidosis and hypercalcemia, which could easily overshadow or mask underlying hypocalcemic conditions. In clinical practice, the coexistence of sarcoidosis with hypoparathyroidism presents unique diagnostic and therapeutic challenges, particularly in the management of calcium homeostasis [6-9].

For instance, the typical presentation of sarcoidosis-associated hypercalcemia might lead clinicians to overlook the possibility of concurrent hypoparathyroidism, which could lead to mismanagement of calcium levels. The presence of hypocalcemia in a sarcoidosis patient should prompt a thorough investigation into possible endocrine disorders, particularly hypoparathyroidism, as this case illustrates. Moreover, the treatment approach for sarcoidosis often involves corticosteroids, which can further complicate calcium regulation by exacerbating hypercalcemia or obscuring underlying hypocalcemia. Corticosteroids affect calcium regulation by inhibiting granulomatous production of 1,25-dihydroxy vitamin D, which reduces calcium absorption from the gut and lowers elevated calcium levels often seen in sarcoidosis. In addition, corticosteroids can also exacerbate hypocalcemia by impairing calcium absorption and increasing renal calcium excretion [10]. Therefore, clinicians should maintain a high index of suspicion for multiple overlapping endocrine disorders in sarcoidosis patients, particularly when standard treatment protocols yield atypical or unexpected outcomes.

Regular monitoring of serum calcium, phosphate, PTH levels, and vitamin D metabolites is essential in patients with sarcoidosis, especially those presenting with signs of calcium dysregulation. This approach ensures that any concurrent disorders, such as hypoparathyroidism, are promptly identified and appropriately managed, thus preventing potential complications arising from improper treatment [11].

The coexistence of sarcoidosis and hypoparathyroidism in the same patient presents a complex pathophysiological scenario that warrants further exploration. Several mechanisms might explain this unusual combination, drawing on existing literature and understanding of both conditions. One possible mechanism involves autoimmune processes. Sarcoidosis is widely recognized as an immune-mediated disorder, and there is growing evidence to suggest that autoimmunity may play a role in its pathogenesis. Hypoparathyroidism, particularly in the absence of a surgical history or identifiable genetic causes, can also have an autoimmune etiology. In this context, it is conceivable that the same or overlapping autoimmune mechanisms might contribute to both sarcoidosis and hypoparathyroidism in the same patient, although this hypothesis requires further investigation [12].

Another potential mechanism could be granulomatous infiltration of the parathyroid glands by sarcoid granulomas. Granulomas, which are the hallmark of sarcoidosis, consist of clusters of activated macrophages, lymphocytes, and other immune cells. These granulomas can infiltrate various organs, leading to organ dysfunction. While granulomatous involvement of the parathyroid glands is rare, it could theoretically impair parathyroid function, resulting in hypoparathyroidism. This hypothesis is supported by cases reported in the literature where sarcoid granulomas have been found in the parathyroid glands, although direct evidence in this case is lacking [13].

Additionally, the interplay between sarcoid granulomas and calcium metabolism presents another layer of complexity. Sarcoid granulomas are known to autonomously produce 1,25-dihydroxyvitamin D, leading to increased intestinal calcium absorption and hypercalcemia. However, in a patient with concurrent hypoparathyroidism, this dysregulated vitamin D metabolism might not fully compensate for the hypocalcemia, leading to the clinical presentation observed in this case. The interaction between these two opposing forces-hypercalcemia driven by sarcoidosis and hypocalcemia due to hypoparathyroidism-creates a delicate balance that can complicate both diagnosis and management [14].

The coexistence of sarcoidosis and primary hypoparathyroidism is rare, and while autoimmune causes are often considered in cases of hypoparathyroidism without surgical history, the potential for familial causes cannot be overlooked. Familial hypoparathyroidism can occur as part of various genetic syndromes, such as autosomal dominant hypocalcemia (ADH), caused by activating mutations in the calcium-sensing receptor (CaSR), or as part of syndromic disorders like DiGeorge syndrome and autoimmune polyendocrine syndrome type 1 (APS-1). Given the absence of a surgical history or neck radiation and no clear autoimmune markers at the time of diagnosis, it would be prudent to investigate potential genetic causes. Testing for mutations in genes associated with familial hypoparathyroidism, such as CaSR, GCM2, or PTH, should be considered to rule out an inherited etiology. In this case, the patient should also be evaluated for autoimmune markers such as anti-parathyroid antibodies, as these may help confirm an autoimmune origin if present [15]. 

In addition to exploring the cause, it is important to recognize the clinical sequelae of long-standing hypoparathyroidism, which can have significant effects on patient outcomes. Chronic hypocalcemia, if untreated or inadequately managed, can lead to neuromuscular complications such as tetany, seizures, and carpopedal spasms. Additionally, long-standing hypoparathyroidism is associated with calcification of basal ganglia, which can result in extrapyramidal symptoms like tremors or movement disorders. Cataracts, dental anomalies, and brittle nails are also common findings in long-term hypocalcemia. Cardiovascular abnormalities, such as prolonged QT interval and arrhythmias, can occur in these patients due to chronic low calcium levels [16].

## Conclusions

This case highlights the rare coexistence of primary hypoparathyroidism and sarcoidosis, presenting with significant hypocalcemia, which is uncommon in sarcoidosis patients who typically exhibit hypercalcemia. The diagnostic challenge arose from the overlapping metabolic disturbances, where the hypocalcemia pointed toward an underlying endocrine disorder rather than being solely attributed to sarcoidosis. Differential diagnoses considered included vitamin D deficiency, chronic kidney disease, and autoimmune hypoparathyroidism. However, normal kidney function, appropriate vitamin D levels, and a lack of surgical history led to the diagnosis of primary hypoparathyroidism, likely autoimmune in origin, with granulomatous infiltration of the parathyroid glands also considered but not confirmed.

Managing this patient requires a multidisciplinary approach due to the complexity of calcium dysregulation and the added effects of corticosteroid therapy for sarcoidosis, which can further obscure or exacerbate underlying hypocalcemia. This case underscores the importance of regularly monitoring calcium, phosphate, and PTH levels in sarcoidosis patients, especially when unexpected biochemical abnormalities are present. Clinicians should be vigilant in recognizing and managing coexisting endocrine disorders to prevent complications, ensuring a comprehensive and tailored therapeutic strategy.
